# Affibody Molecules as Targeting Vectors for PET Imaging

**DOI:** 10.3390/cancers12030651

**Published:** 2020-03-11

**Authors:** Vladimir Tolmachev, Anna Orlova

**Affiliations:** 1 Department of Immunology, Genetics and Pathology, Uppsala University, 75185 Uppsala, Sweden; 2 Research Centrum for Oncotheranostics, Research School of Chemistry and Applied Biomedical Sciences, Tomsk Polytechnic University, 634050 Tomsk, Russia; anna.orlova@ilk.uu.se; 3 Department of Medicinal Chemistry, Uppsala University, 75183 Uppsala, Sweden; 4 Science for Life Laboratory, Uppsala University, 75237 Uppsala, Sweden

**Keywords:** PET, affibody molecules, HER2, EGFR, molecular imaging, radiolabeling

## Abstract

Affibody molecules are small (58 amino acids) engineered scaffold proteins that can be selected to bind to a large variety of proteins with a high affinity. Their small size and high affinity make them attractive as targeting vectors for molecular imaging. High-affinity affibody binders have been selected for several cancer-associated molecular targets. Preclinical studies have shown that radiolabeled affibody molecules can provide highly specific and sensitive imaging on the day of injection; however, for a few targets, imaging on the next day further increased the imaging sensitivity. A phase I/II clinical trial showed that ^68^Ga-labeled affibody molecules permit an accurate and specific measurement of HER2 expression in breast cancer metastases. This paper provides an overview of the factors influencing the biodistribution and targeting properties of affibody molecules and the chemistry of their labeling using positron emitters.

## 1. Introduction

Radionuclide molecular imaging permitting non-invasive quantitative visualization of molecular targets is an attractive alternative to biopsy-based methods for stratifying patients for targeted therapies [1]. The use of positron emission tomography (PET) is a preferable method for molecular imaging because it provides a better spatial resolution, registration efficiency, and accuracy of activity quantification compared to single photon emission computed tomography (SPECT) [2].

Currently, the most common approach used to visualize molecular targets is immunoPET, which is a methodology based on the labeling of therapeutic monoclonal antibodies with long-lived positron emitting nuclides, including ^64^Cu (T_½_ = 12.7 h), ^89^Zr (T_½_ = 78.4 h), and ^124^I (T_½_ = 100.2 h) [3,4]. The feasibility of such an approach has been demonstrated in a number of clinical studies [5,6,7]. Accumulated clinical experience has enabled the identification of several major issues in the routine clinical use of full-length immunoglobulin G (IgG) as imaging probes; these issues include slow extravasation and accumulation in tumors, slow clearance from blood and unspecific compartments, and unspecific uptake in target-negative tumors due to the enhanced permeability and retention (EPR) effect. To obtain an acceptable contrast and sensitivity, clinical imaging must be performed 4-7 days after injection [5,6,7]. Nevertheless, the unspecific tumor accumulation of IgG is associated with a risk of false-positive diagnostics [8]. Therefore, it is very likely that immunoPET will remain a valuable tool for the development of antibody-based therapeutics, but the prospects of its translation into day-by-day clinical practice are unclear.

Empirical data suggest that a reduction in the size of targeting proteins permits the ability to obtain a high imaging contrast earlier than several days after the injection [9]. Indeed, the use of a single-domain antibody (sdAb) with a molecular weight of 12–15 kDa permits the development of imaging probes with a tumor-to-blood ratio of 10–30 at 1 h after injection in murine models [10]. Mathematical modeling predicts that a further size reduction would enable an increase in tumor accumulation if the affinity of imaging probes was high enough [11,12]. Thus far, the development of high-affinity immunoglobulin-based targeting probes smaller than a sdAb remains very challenging. Nevertheless, this is possible using engineered non-immunoglobulin scaffold proteins [13].

## 2. Engineered Scaffold Protein-Based Imaging Probes: Affibody Molecules

Antibodies spectacularly exemplify natural affinity proteins capable of specific binding to a large variety of molecular motifs. Nevertheless, there is a broad repertoire of natural binding proteins, which might be utilized to develop novel affinity ligands. The key feature of such binders is the presence of a robust skeleton called a scaffold, which ensures the stable positioning of variable amino acids and minimizes the entropy penalty. Large combinatorial libraries, created by the randomization of variable amino acids, permit the selection of proteins binding with a high affinity and specificity to desirable molecular structures. Such binders are called engineered scaffold proteins (ESPs). ESPs are increasingly applied as a molecular recognition moiety for targeting therapeutics and are considered a complement to or substitution of monoclonal antibodies. We recommend several excellent review papers for a more detailed description of ESPs [14,15,16,17].

**Figure 1:**
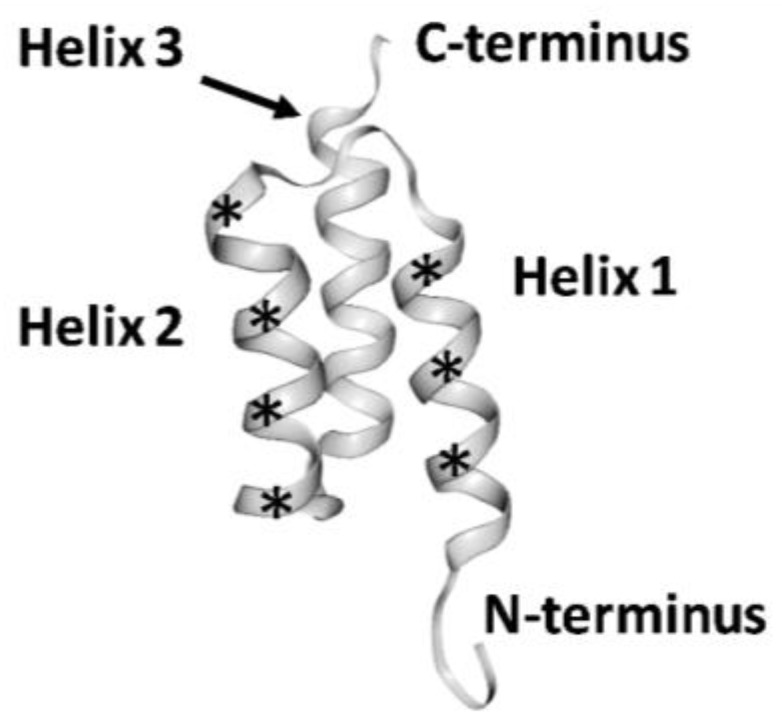
Structure of an affibody scaffold. Asterisks mark the segments where randomized amino acids are located. Image was obtained from the RCSB Protein Data Bank (rcsb.org) of PDB ID 2KZJ showing HER2-.

To the best of our knowledge, the first type of scaffold protein, which was evaluated in vivo for radionuclide imaging, was that of affibody molecules [18,19]. Affibody molecules utilize a cysteine-free three-helical scaffold (Figure 1) of a modified B-domain of protein A (58 amino acids, 7 kDa) [20]. A combinatorial library has been created by the randomization of thirteen amino acids located on helices one and two and normally involved in the binding of the Fc domain of immunoglobulins. Phage-display selection enables the generation of affibody molecules with affinities in the nanomolar range [21]. Affinity maturation permits a further increase of the affinity from a few nM to a few pM. High-affinity affibody binders have been selected for potential cancer-associated molecular targets such as human epidermal growth factor receptor type 2 (HER2) [22], epidermal growth factor receptor (EGFR or HER1) [23], human epidermal growth factor receptor type 3 (HER3) [24], insulin-like growth factor-1 receptor (IGF-1R) [25], platelet-derived growth factor receptor β (PDGFRβ) [26], vascular endothelial growth factor receptor 2 (VEGFR2) [27], programmed death-ligand 1 (PD-L1) [28], and carbonic anhydrase IX (CAIX) [29].

Several features of affibody molecules make them potentially attractive as targeting vectors for imaging probes:

- Their small size ensures rapid extravasation and diffusion in the extracellular space, providing efficient localization in tumors. Unbound affibody molecules are rapidly excreted from blood via glomerular filtration, and this reduces the background.
- Affibody molecules can be selected to have a high affinity and specificity to a desirable molecular target.
  *These Features Together Safeguard the Rapid Acquisition of a High Imaging Contrast and Determine the High Sensitivity of Molecular Imaging*
  - Small proteins (less than 45 kDa) do not accumulate in tumors by the EPR effect [30]. This reduces the risk of false-positive diagnostics.
  - Affibody molecules are proteolytically, chemically, and thermally stable and refold within 3 µs with a high fidelity after thermal or chemical denaturation [31]. This permits the use of high temperatures (up to 90–95 °C), which enables rapid labeling using macrocyclic chelators [32,33], a broad range of pH values (from 3.6 to 11.0) [32,33,34,35], and lipophilic organic solvents for intermediate purifications [36], without losing specificity and affinity.
  - Affibody molecules do not contain cysteines, and their folding is independent of disulphide bridges. This allows the use of large amounts of oxidants (e.g., during electrophilic iodination) or reductants (e.g., during labeling with ^99m^Tc or ^186/188^Re), without the risk of denaturation.

Affibody molecules can be produced recombinantly in prokaryotic hosts or by peptide synthesis. This makes their production appreciably more cost-efficient than the production of antibodies.Affibody molecules can be labeled in a site-specific manner. With the use of peptide synthesis, chelators, unnatural amino acids, prosthetic groups, or pharmacokinetic modifiers might be easily introduced at the N-terminus [35,36,37,38,39]. The use of an orthogonal protection scheme permits the selective deprotection of a preselected lysine and amino group-directed coupling of a chelator or prosthetic group in any position of the scaffold [40,41]. Another approach is the incorporation of a unique cysteine into a recombinantly produced protein and the use of thiol-specific chemistry for the conjugation of a chelator or prosthetic group. *The Use of Site-Specific Coupling/Labeling Provides a Homogenous Product with a Reproducible Biodistribution and Targeting Properties*.

The incorporation of a cysteine is also possible during peptide synthesis, which enables the thiol-directed coupling of chelators [42]. In that case, the same tracer could be produced using both recombinant production and peptide synthesis, but would have the same properties. To make affibody molecules more suitable for peptide synthesis, the scaffold was redesigned [43]. β-Branched amino acids (V, I, and T) and amino acids prone to side reactions during peptide synthesis (D, N, and H) were replaced. This resulted in an increase in the peptide synthesis yield from 13% to 22%. Unexpectedly, the melting point of the new scaffold increased by 12 °C. Despite the substitution of 11 water-exposed amino acids, the pattern of biodistribution of the second generation of affibody molecules remained the same [44,45].

## 3. Biodistribution and Targeting Features of Radiolabeled Affibody Molecules

The biodistribution and targeting patterns of radiolabeled affibody molecules are determined by four major features:

- Small size;
- Slow internalization after specific binding to a molecular target;
- High reabsorption in proximal tubules;
- Elevated hepatic uptake and/or hepatobiliary exertion after modifications, increasing the overall or local lipophilicity.

The small size is essential for rapid extravasation and localization in tumors. Typically, the maximum tumor uptake in murine models is reached within 30 min after injection [44,46]. Nevertheless, some time is required for clearance from blood and normal tissues to reach a good imaging contrast. Usually, the unrestricted glomerular filtration of small affibody molecules results in rapid blood clearance and provides excellent contrast within 2–4 h after injection. The only exception is the case when there is a noticeable expression of molecular targets in normal tissues. In this case, another phenomenon—slow internalization after binding to a molecular target—might play a critical role. Slow internalization (less than 30% activity internalized by cancer cells within 24 h in vitro) was found for monomeric forms of affibody molecules binding HER2 [47], EGFR [23], HER3 [24], and CAIX [29]. EGFR is expressed by hepatocytes, and radiolabeled anti-EGFR affibody molecules are actively taken up by the liver [23,48]. Due to the slow internalization, at least some radiolabeled affibody molecules remain bound to receptors on hepatocyte membranes for a while and dissociate when the blood concentration decreases due to clearance. Therefore, the liver acts as a depot, and the blood clearance of anti-EGFR affibody molecules is slower than clearance of affibody molecules to other targets [23,48,49,50]. An optimal imaging time might be 24 h after injection in this case. A similar effect has been observed for HER3-targeting affibody molecules [51].

Another feature of affibody molecules is the very efficient reabsorption in the proximal tubules of kidneys. This feature has been observed for all binders, independent of the molecular target [23,24,25,26,27,29,37]. Therefore, it is reasonable to suppose that the reabsorption is determined by the scaffold rather than by a binding site. Studies with megalin knock-out mice demonstrated that megalin is not involved in the renal reabsorption of affibody molecules [52]. A recent study showed that the use of lysine, gelofusine, diuretics mannitol and furosemide, and the organic anion transporter blocker probenecid, is inefficient for the reduction in renal reabsorption of affibody molecules [53]. The inhibition of ATP-mediated endocytosis by sodium maleate and fructose resulted in an approximately two-fold decrease in the renal uptake of anti-HER2 affibody molecules [53]. In the case of residualizing radiometal labels, the renal uptake of activity might exceed the tumor uptake by 10–20-fold [23,24,25,26,27,29,37]. Initially, there was concern that the high renal uptake of activity would lead to reconstruction artefacts obscuring metastases in the lumbar area. However, clinical studies have demonstrated that it is possible to visualize metastases not only in the lumbar vertebrae, but also in adrenals, using radiometal-labeled affibody molecules [53,54]. Nevertheless, the high renal uptake contributes to an absorbed dose burden to patients.

**Figure 2:**
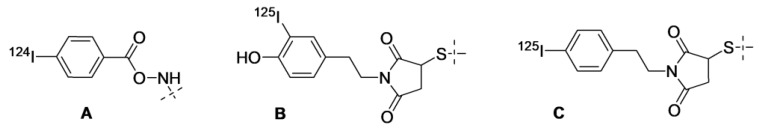
Structures of non-residualizing radioiodine labels provided by the labeling of affibody molecules using PIB (**A**), IHPEM (**B**), and IPEM (**C**).

**Figure 3:**
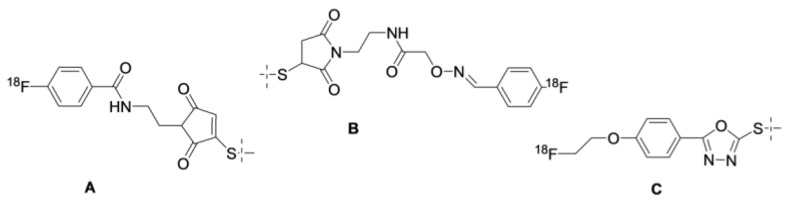
Structures of non-residualizing radiofluorine labels provided by the labeling of affibody molecules using FBEM (**A**), FBA (**B**), and FPOS (**C**).

One possible solution for the issue of renal uptake is based on the slow internalization of affibody molecules after binding to cancer cells. In this case, the use of residualizing labels has a moderate contribution to an overall retention of activity in tumors. On the other hand, the internalization of affibody molecules after renal reabsorption is rapid. This results in the fast transport of affibody molecules to lysosomes of proximal tubules and a prompt release of radiometabolites from proximal tubule cells. This phenomenon is typical for radiohalogen labels. The first experiments with affibody molecules labeled using [^125^I]-4-iodobenzoate (Figure 2) already demonstrated that renal uptake is reduced much more rapidly than tumor uptake, and a few hours after injection, tumor uptake exceeds the radioactivity concentration in kidneys [22]. The release of activity from kidneys depends on the radioiodine-bearing prosthetic group and the position of its coupling to an affibody molecule. Therefore, the clearance of activity from kidneys is quicker when using 3-iodo-((4-hydroxyphenyl) ethyl)maleimide [55] and 3-bromo-((4-hydroxyphenyl)ethyl)maleimide [18] (Figure 2) than using 4-iodobenzoate and 4-bromobenzoate. The use of iodophenetylmaleimide (Figure 2) as a precursor resulted in an even more rapid clearance of activity from kidneys [55]. Similarly, low renal uptake was associated with the use of non-residualizing radiofluorine labels N-2-(4-[^18^F]fluorobenzamido)ethyl] maleimide ([^18^F]-FBEM) [56,57], [^18^F]-N-(4-fluorobenzylidene)oxime ([^18^F]-FBO) [38,46,58], and [^18^F]-fluoro-phenyloxadiazole methylsulfone ([^18^F]-FPOS) [59] (Figure 3). In all cases, there was only a minor decrease in tumor uptake in comparison with the use of residualizing labels. It is important to recall that renal radiometabolites may be released into blood and, possibly, redistributed to other tissues, before excretion. This might result in decreased tumor-to-blood and tumor-to-organ ratios due to elevated levels of blood-borne radioactivity [60].

It should be noted that non-residualizing labels are often associated with lipophilic prosthetic groups. In this case, the elevated hepatic uptake and/or hepatobiliary excretion of a tracer or its radiometabolites might be expected. Our review [61] demonstrates this data for affibody molecules labeled with single-photon emitters. The same effect was observed for an anti-HER2 affibody molecule radiofluorinated at the C-terminus using several different methods [58]. In that case, labeling using hydrophilic [^18^F]-AlF-NOTA was associated with a low level of hepatobiliary excretion. In the case of [^18^F]-FBO labeling, 40–50% of the activity was found in the intestines and their contents. A quite appreciable level of hepatobiliary excretion was also observed in the case of the labeling of affibody molecules at the C-terminus using [^18^F]-FPOS [59]. Interestingly, we demonstrated that the use of a negatively charged triglutamyl (EEE) linker enabled a substantial reduction in hepatobiliary excretion when anti-HER2 affibody molecules were labeled using [^18^F]-FBO [38]. It is worth investigating if the same effect takes place for affibody molecules with other specificities or labeled at different positions. This issue is essential because an elevated hepatic uptake complicates the imaging of liver metastases, which are frequent in different cancers [62]. A high level of hepatobiliary excretion is a problem for imaging extrahepatic abdominal metastases.

## 4. Affinity and Dimerization

**Figure 4:**
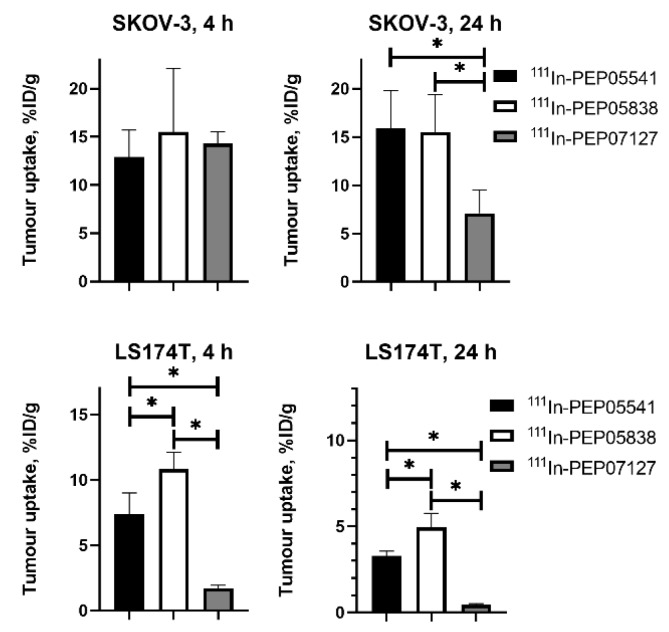
Influence of the affinity of affibody molecules on the accumulation in tumor xenografts with high (SKOV-3) and low (LS174T) human epidermal growth factor receptor type 2 (HER2) expression at different time points. Asterisk (*) indicates significant (*p* < 0.05) difference. Data are taken from [63].

An important consequence of slow internalization means that tumor activity retention depends on the slow dissociation of an affibody molecule from a target on a cancer cell surface. Therefore, a sufficiently high affinity is a precondition for high-contrast imaging. To estimate the level of affinity, which is required for successful imaging, a set of ^111^In-labeled affibody molecules was evaluated in mice bearing xenografts with high (SKOV-3, 1.63 × 10^6^ receptors per cell) and low (LS174T, 3.9 × 10^4^ receptors per cell) HER2 expression levels. Affibody molecules PEP05541 (K_D_ = 116.7 ± 0.1 pM), PEP05838 (K_D_ = 157 ± 4 pM), and PEP07127 (K_D_ = 3804 ± 178 pM) were evaluated [63]. It was shown (Figure 4) that in tumors with a high expression level, the uptake 4 h after injection was independent of affinity up to 4 nM. However, the tumor activity retention 24 h after injection was significantly better for affibody molecules with an affinity of 110–160 pM.

In the case of low expression levels, the high affinity affibody molecules provided a much higher tumor uptake 4 h after injection, and the difference was even more pronounced at 24 h. Based on that study, we can state that affinity in the single digit nanomolar range is sufficient for imaging targets with a high expression level (over 10^6^ target molecules per cell), but a subnanomolar affinity is desirable in the case of lower expression (<10^5^ target molecules per cell).

**Figure 5:**
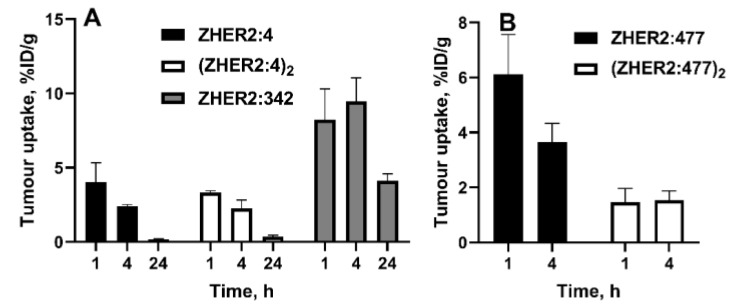
Effect of the dimerization of affibody molecules ZHER2:342 (**A**) and ZHER2:477 (**B**) on the uptake in HER2-expressing xenografts in mice. Index 2 in designations (ZHER2:4)_2_ and (ZHER2:477)_2_ shows that these affibody constructs contain two monomeric units fused head-to-tail. Data are taken from [22,72].

A high affinity of affibody molecules might be achieved by affinity maturation [22,64,65,66]. This process requires appreciable competence and might take some time. The use of dimerization might be considered an attractive alternative to the affinity maturation. The avidity effect in this case might provide up to an order of magnitude higher affinity [67,68]. Moreover, the feasibility of radionuclide imaging using the dimeric form of affibody molecules has been demonstrated [18,19,69,70]. However, the results of a direct comparison [22] have demonstrated that a radioiodinated dimeric form of the anti-HER2 affibody molecule (ZHER2:4)2 (K_D_ = 3 nM) does not provide higher tumor uptake than its monomeric form ZHER2:4 (K_D_ = 50 nM), despite the appreciably higher affinity of the dimer (Figure 5A). At the same time, a high affinity monomeric form ZHER2:342 (K_D_ = 0.029 nM) provided an appreciably higher tumor uptake. However, further dimerization of ZHER2:342 resulted in a decrease in tumor uptake [71]. A decrease in tumor uptake was observed for the dimeric form of another clone of the anti-HER2 affibody molecule, ZHER2:477, which was labeled using [^64^Cu]-DOTA (Figure 5B) [72] and [^18^F]-FBO [46]. In both of these cases, (ZHER2:477)_2_ had a higher affinity than ZHER2:477. A similar effect was observed for the EGFR-binding affibody ZEGFR:1907 labeled with ^125^I and ^111^In [73].

The effect of dimerization might be explained by a decrease in the extravasation rate with an increase in the size. Overall, it is apparent that an increase in the affinity of affibody molecules should be pursued by affinity maturation and not dimerization.

## 5. Injected Mass and Molar Activity

**Figure 6:**
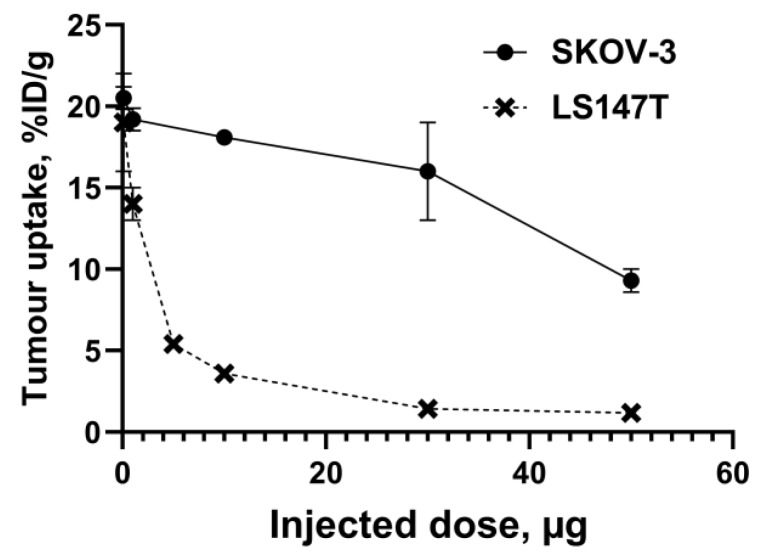
Influence of the injected affibody mass on the uptake of the [^111^In]-In-DOTA-ZHER2:342 affibody molecule in tumor xenografts with high (SKOV-3) and low (LS174T) levels of HER2 expression.

The imaging of HER2 is necessary for the discrimination of tumors with a 3+ (eligible for trastuzumab or lapatinib treatment) from 2+ (not eligible for such treatment) level of expression [74]. However, breast cancer tumors with 2+ expression have a noticeable number of HER2 receptors on their surface [75], and we have to be able to discriminate between tumors with high and low expression levels. Experiments with mice bearing xenografts with high and low levels of HER2 demonstrated that when an injected mass of anti-HER2 affibody molecules is low (0.1 µg (0.014 nmol)/mouse), the tumor uptake is equally high for tumors with both expression levels (Figure 6) [76]. However, an increase in the injected mass reduces the uptake in tumors with low expression, while the uptake reduction in tumors with high expression is not dramatic. The results of this experiment have been confirmed in a clinical study [54,77]. 

The optimal injected mass is directly connected with the desirable molar activity of affibody-based imaging probes for clinical translation. A clinical study concerning the imaging of HER2 using ^68^Ga-labeled ABY-025 affibody molecules demonstrated that an injected activity of 212 ± 46 MBq permits a good imaging quality up to 4 h after injection [54] and is associated with an acceptable effective dose (5–6.4 mSv) [77]. In that study, a molar activity of about 3.8 MBq/µmol (injected mass 56 µmol/427 mg) provided better discrimination between metastases with high and low HER2 expression than a molar activity of 21 MBq/µmol (injected mass 10 µmol/78 mg). We suppose that a molar activity of 3–4 MBq/µmol is close to optimal for HER2 imaging using affibody molecules labeled with ^68^Ga and, possibly, ^18^F.

The imaging of HER2 is the simplest case, as the expression of this target in normal adult tissues is low. Some other targets (e.g., EGFR, HER3, and IGF-1R) are appreciably expressed in normal tissues. In particular, liver expression is troublesome, as it not only causes low contrast in the imaging of frequently occurring hepatic metastases, but might also cause the sequestering of an imaging probe and reduce its tumor uptake in clinics [78]. It has been demonstrated that it is possible to find an optimal mass of unlabeled affibody molecules to be co-injected with a labeled probe, which blocks receptors in liver and some other normal tissues, but does not reduce the tumor uptake. This has been demonstrated for affibody molecules for the imaging of EGFR [23,57,73], HER3 [24], and IGF-1R [25]. Assuming the same scaling factor as for the imaging of HER2, the optimal injected mass would be around 1.3, 0.065, and 0.035 mg, corresponding to molar activities of 1.4, 28, and 57 MBq/µmol for ^68^Ga-labeled EGFR, HER3, and IGF-1R, respectively. It has to be noted that the affibody molecules that were used in these studies have an equal affinity to human and murine receptors. However, the expression level of these receptors might be different in mice and humans. Therefore, our values can only be considered as starting points in clinical dose-finding studies.

## 6. Labeling of Affibody Molecules with Positron-Emitting Radionuclides

Two positron emitting nuclides are the most interesting for the clinical translation of affibody molecules: ^18^F (T _½_ = 109.8 min) and ^68^Ga (T _½_ = 67.6 min). The half-lives of both of these nuclides are sufficient for imaging several hours after injection, and this is compatible with the rapid pharmacokinetics of affibody molecules. Fluorine-18 is a favorite of the radiopharmaceutical industry, since centers capable of the multicurie production of [^18^F]F^−^ have been installed for manufacturing [^18^F]-FDG, and delivery logistics have been established. Gallium-68 can be produced from a long-lived ^68^Ge/^68^Ga generator, even at PET centers without its own cyclotron. In addition, ^68^Ga can be produced by cyclotrons using easy-to-operate liquid targets [79].

### 6.1. Fluorine-18

Despite great progress in radiofluorination, labeling of proteins and peptides remains challenging due to the multistep synthesis of precursors and prosthetic groups, leading to low yields and/or a limited stability of labels [80].

Kramer-Marek and co-workers have reported site-specific labeling of the anti-HER2 affibody molecule ZHER2:342 with a C-terminal cysteine using N-(2-(4-[^18^F]-fluorobenzamido) ethyl) maleimide ([^18^F]FBEM) (Figure 3A) [56]. The multistep synthesis with intermediate HPLC purification resulted in a radiochemical yield of 6.5 ± 2.2% after a 2-h long procedure. This tracer demonstrated an excellent tumor-to-blood ratio (69 ± 27) 4 h post-injection (pi). The use of a non-residualizing label resulted in a low retention of activity in the kidneys and liver (tumor-to-liver ratio of 18). However, the tumor-to-bone ratio was moderate (5 ± 2.6), possibly due to the release of radiometabolites after renal catabolism. The same methodology has been applied for labeling an anti-EGFR affibody molecule, Cys-ZEGFR:1907, with an N-terminal cysteine [57]. The radiochemical yield remained rather low (10%, decay-corrected) after a 3-h synthesis. Despite the use of a non-residualizing label, the high liver uptake resulted in tumor-to-liver ratio of 1.25. It is possible that this is the result of an additional lipophilicity on the N-terminus due to labeling.

A more efficient approach for the radiofluorination of peptides is based on the reaction of 4-[^18^F]-fluorobenzaldehyde ([^18^F]-FBA) with an oxoamine, resulting in oxime formation [81]. The labeling of the affibody molecule ZHER2:477 or its dimer was performed by the maleimido-mediated coupling of an oxoamine to the C-terminus, with a subsequent reaction with [^18^F]-FBA (Figure 3B) [46,82]. The overall yield can be estimated as 15–20% (decay-corrected). Further optimization of this approach permitted labeling of the anti-HER2 affibody molecule ZHER2:2981-C (GE-226) with an overall non-decay corrected yield of 30% using FASTlab [83,84].

**Figure 7:**
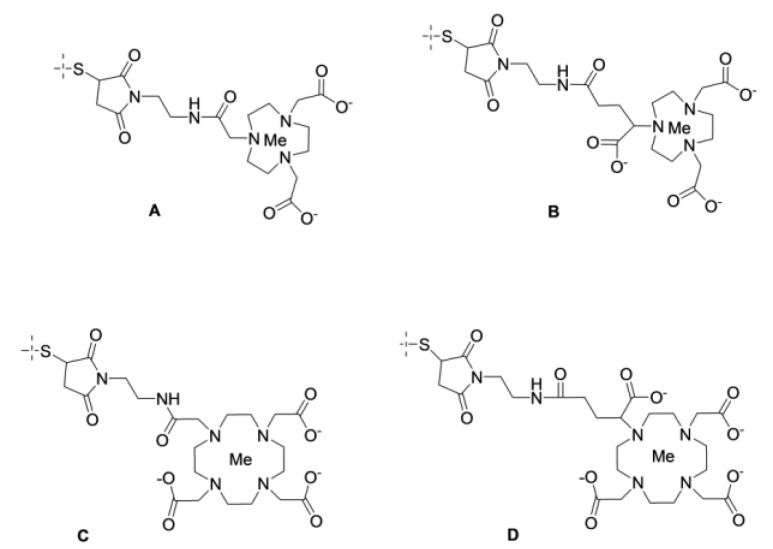
Macrocyclic chelators NOTA (**A**), NODAGA (**B**), DOTA (**C**), and DOTAGA (**D**) conjugated to cysteine using maleimido derivatives.

Another efficient approach for improving the radiofluorination yield is based on the formation of [^18^F]aluminium monofluoride ([^18^F]-AlF), with subsequent complexation by derivatives of the NOTA chelator [85]. To demonstrate the feasibility of the method for labeling affibody molecules, the anti-HER2 affibody molecule ZHER2:2395-C was conjugated with a maleimido derivative of NOTA (Figure 7A) at the C-terminus [36]. The chelation of [^18^F]AlF was performed in acetate buffer at pH 4.0 and 90 °C for 15 min, with subsequent purification using an HLB cartridge and buffer exchange using an NAP-5 size-exclusion column. The overall radiochemical yield was 21 ± 6% after the 40-min procedure. The refolding capacity of affibody molecules provided a preserved affinity after labeling in such harsh conditions. [^18^F]-AlF-NOTA-ZHER2:2395 demonstrated excellent tumor targeting 4 h pi (tumor-to-blood ratio of 145 ± 24), but the bone uptake was twice as high as that of its ^111^In-labeled counterpart, which indicates a somewhat lower chelate stability. This approach has been applied to label the HER2-binding affibody molecules NOTA-CGGGRDN-ZHER2:234 [86], and NOTA-ZHER2:2891-C [58], PD-L1-binding NOTA-ZPD-L1_1 [28], and EGFR-binding NOTA-ZEGFR:1907 [87] and NOTA-ZEGFR:03115 [88], providing radiochemical yields in the range of 10–15% after a 40-min procedure.

**Figure 8:**
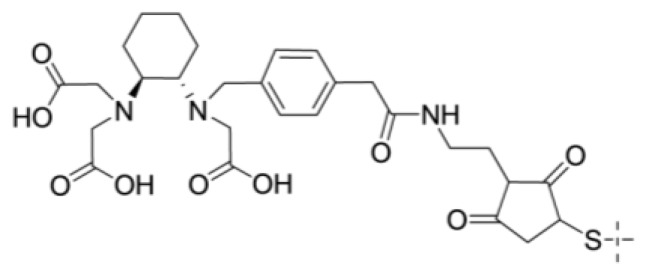
H_3_RESCA conjugated to a thiol group.

A new acyclic chelator known as RESCA (Figure 8) has been developed for the [^18^F]-AlF-labeling of heat-sensitive proteins at moderate temperatures [89]. The labeling of ZHER2:2891 (PEP04314) with [^18^F]-AlF was performed at 37 °C for 15 min and resulted in a radiochemical yield of 20 ± 7%. In rhesus monkeys, [^18^F]-AlF-RESCA-PEP04314 had a biodistribution similar to the biodistribution of [^18^F]-AlF-NOTA-PEP04314, but with a somewhat elevated bone uptake, which might indicate a lower label stability.

**Figure 9:**
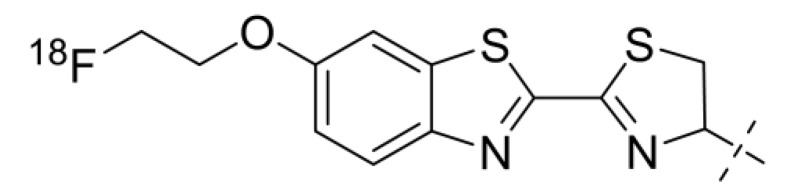
^18^F-labeled 2-cyanobenzothiazole ([^18^F]-F-CBT)-label coupled to an N-terminal cysteine.

Su and co-workers evaluated the thiol-directed labeling of ZEGFR:1907 affibody molecules with C-terminal cysteine using ^18^F-labeled 2-cyanobenzothiazole ([^18^F]-F-CBT) (Figure 9) [87]. A two-step, two-pot 120-min procedure resulted in a decay-corrected radiochemical yield of 41%. A direct comparison of [^18^F]F-CBT-ZEGFR:1907 and [^18^F]AlF-NOTA-ZEGFR:1907 in mice bearing A431 xenografts demonstrated that both variants had a similar tumor uptake, but the renal uptake of [^18^F]F-CBT-ZEGFR:1907 was 14-fold and hepatic uptake 4.3-fold lower than the uptake of [^18^F]AlF-NOTA-ZEGFR:1907. This is characteristic of non-residualizing labels. However, the bone uptake of [^18^F]-F-CBT-ZEGFR:1907 was 7.4-fold higher and exceeded the tumor uptake. In combination with a very high level of hydrophilic radiometabolites in blood, this suggests that [^18^F]fluoride is released from the [^18^F]F-CBT label, possibly during renal and hepatic catabolism. Therefore, this label is not suitable for affibody molecules.

**Figure 10:**
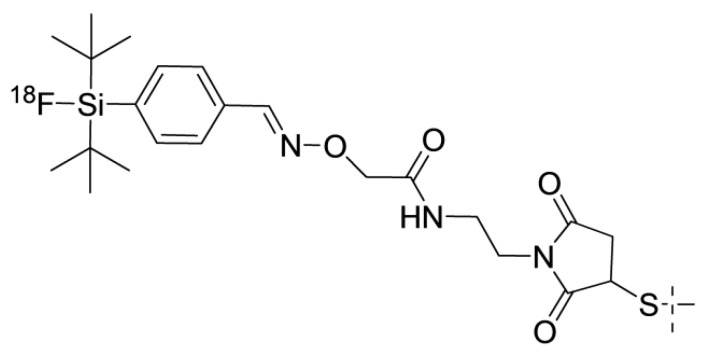
[^18^F]*p*-(di-tert-butyl-fluorosilyl)benzaldehyde ([^18^F]-F-SiFA)-label coupled to a C-terminal cysteine.

Glaser and co-workers [58] performed a comparative evaluation of ZHER2:2891-C affibody molecules labeled using [^18^F]AlF-NOTA, [^18^F]FBA, and the silicon-fluoride acceptor [^18^F]*p*-(di-tert-butyl-fluorosilyl)benzaldehyde ([^18^F]-SiFA-A). [^18^F]-SiFA-labeling (Figure 10) was performed according to the approach proposed by Schirrmacher and co-authors [90] and provided an excellent yield of 38 ± 2%. However, the injection of [^18^F]-SiFA- ZHER2:2891-C resulted in a substantial increase in the bone uptake with time, indicating a defluorination of the tracer. The use of a non-residualizing [^18^F]FBA label provided a much lower renal uptake than that of [^18^F]AlF-NOTA, but 40–50% of the activity was excreted via bile, which might complicate the detection of extrahepatic abdominal metastases.

[^18^F]-FPOS (fluorophenyloxadiazole methylsulfone) (Figure 3C) can be obtained in a single step within 10 min, with a yield of 27 ± 6% [59]. This precursor was site-specifically conjugated to cysteine of ZHER2:2395-C with a yield of 40% [59]. [^18^F]-FPOS-ZHER2:2395-C was capable of the specific visualization of HER2-expressing xenografts in mice, but high activity levels in the gall bladder and intestines indicated a high degree of hepatobiliary excretion.

Overall, [^18^Al]-AlF-NOTA-, [^18^F]-FBA-, and [^18^F]-FPOS chemistry provide the fluorination of affibody molecules with yields compatible with clinical translation. However, the use of [^18^F]-FBA and [^18^F]-FPOS is associated with undesirable hepatobiliary excretion. It is worth evaluating if the application of hydrophilic linkers (e.g., EEE) before the C-terminal cysteine can suppress this excretion pathway.

### 6.2. Gallium-68

**Figure 11:**
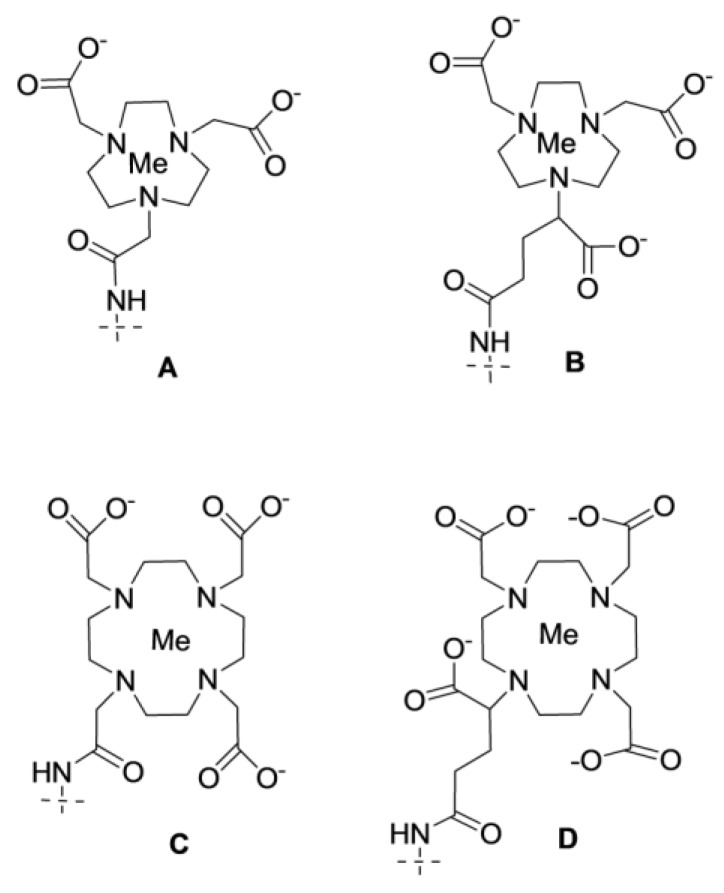
Macrocyclic chelators NOTA (**A**), NODAGA (**B**), DOTA (**C**), and DOTAGA (**D**) conjugated to the amino group of synthetic affibody molecules.

The excellent refolding of affibody molecules after thermal denaturation permits efficient and stable high-temperature labeling with ^68^Ga using macrocyclic chelators (Figure 7 and Figure 11). The target specificity is preserved in this case, as has been demonstrated by the labeling of the synthetic anti-HER2 ZHER2:342 affibody molecule with the DOTA chelator conjugated to an N-terminal amino group (Figure 11C) [91]. Labeling at 90 °C resulted in a radiochemical yield of 98.6 ± 0.7% within 10 min. Hydrophilicity of the chelator-metal complex was associated with suppressed hepatobiliary excretion, but renal excretion was followed by nearly complete reabsorption and an efficient retention of activity in the kidneys. This pattern is characteristic of nearly all radiometal-labeled affibody molecules.

The incorporation of a radiometal-chelator complex modifies the local charge and lipophilicity of the surface of a protein. This can affect off-target interactions with normal tissues and binding to blood proteins. It was shown for ^111^In- and ^99m^Tc-labeled affibody molecules that different positions of a chelator in the affibody scaffold have an apparent influence on the imaging contrast [40,41]. The effect of a label position for the ^68^Ga-labeled variant was studied for the synthetic anti-HER2 ZHER2:S1 affibody molecule, which was conjugated with DOTA via an amine bond at the N-terminus (A^1^), in the middle of helix 3 (K^50^), or at the C-terminus (K^58^) [92]. All variants had a similar affinity to HER2 in the range of 75–95 pM. It was found that positioning of the label in the middle of helix 3 offered an appreciably worse contrast than that in other variants. For example, the tumor-to-blood ratio was nearly three-fold lower. The majority of the tumor-to-organ ratios were equal to the other two variants, but [^68^Ga]Ga-(DOTA-A^1^)-ZHER2:S1 provided a significantly higher tumor-to-lung ratio than [^68^Ga]Ga-(DOTA-K^58^)-ZHER2:S1.

A comparative evaluation of the synthetic affibody ^68^Ga-labeled at the N-terminus using NOTA, NODAGA, and DOTA (Figure 11A–C) showed that [^68^Ga]Ga-NODAGA-ZHER2:S1 had the highest tumor-to-blood ratio (60 ± 10) at 2 h pi in comparison with both [^68^Ga]Ga-DOTA-ZHER2:S1 (28 ± 4) and [^68^Ga]Ga-NOTA-ZHER2:S1 (42 ± 11). The same variant had the highest tumor-to-liver ratio [33]. These data demonstrated that both the charge and structure of the chelator-radiometal complex influence the biodistribution of the affibody molecule. To confirm this, the biodistribution of synthetic ZHER2:2891 (with an optimized scaffold) labeled with ^68^Ga using DOTA (neutral complex) and DOTAGA (single negative charge of the complex) (Figure 11D) was evaluated [93]. An increase in the negative charge at the N-terminus of [^68^Ga]Ga-DOTAGA-ZHER2:2891 was associated with a two- to three-fold increase in the tumor-to-blood, tumor-to-lung, tumor-to-liver, and tumor-to-bone ratios. These (and a number of other) studies have demonstrated that the labeling strategy (i.e., selection of an optimal position and structure of a label) has a strong influence on the contrast and thus on the sensitivity of imaging using affibody molecules.

Site-specific labeling of a recombinantly produced ZHER2:2891-C (ABY-025) affibody molecule via DOTA conjugated to a C-terminal cysteine (Figure 7C) enabled a yield of >90% after 15 min at 80 °C and excellent targeting properties [45]. In a clinical phase I/II study, this tracer demonstrated high-contrast imaging in HER2-expressing tumors and enabled the discrimination between breast cancer metastases with high and low HER2 expression levels [54]. [^68^Ga]-Ga-ABY-025 can be automatically produced on the Modular-Lab PharmTrace synthesis platform (Eckert & Ziegler), with a non-decay corrected radiochemical yield of approximately 40% [94]. Conjugation of the maleimido derivative of DOTA also permitted the rapid and efficient ^68^Ga-labeling of PDGFRβ-binding ZPDGFRβ:09591 and resulted in a PET tracer providing a high imaging contrast 2 h pi [95]. Maleimido derivatives of the triaza chelators NOTA and NODAGA (Figure 7A,B) were successfully used for ^68^Ga labeling of the anti-HER3 ZHER3:0689 affibody molecule [96,97]. It was found that the hepatic uptake and hepatobiliary excretion decreased with an increase in the negative charge of the chelator-metal complex. Furthermore, this effect was further enhanced by the placement of the HEHEHE sequence at the N-terminus.

**Figure 12:**
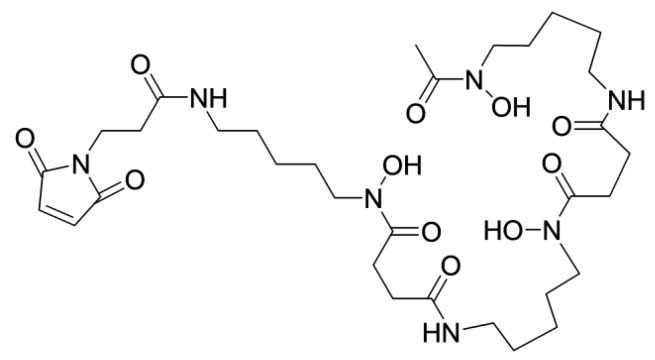
Structure of the deferoxamine (DFO)-maleimido derivative.

**Figure 13:**
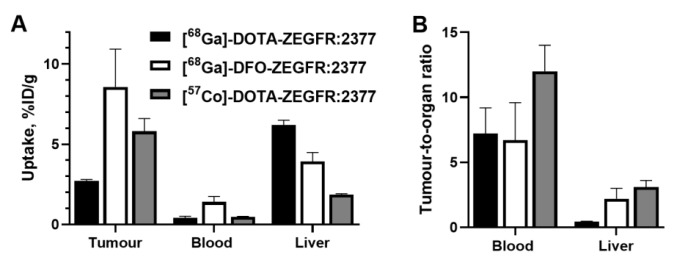
Uptake (**A**) and tumor-to-organ ratios (**B**) for ZEGFR:2377 affibody molecules labeled using [^68^Ga]-DOTA-, [^68^Ga]-DFO-, and [^57^Co]-DOTA-radionuclide/chelator combinations in mice bearing A431 xenografts 3 h after injection. Data from [99,101].

The acyclic siderophore-derived chelator deferoxamine (DFO) is suitable for the labeling of proteins with ^68^Ga [98]. Site-specific conjugation of a maleimido derivative of DFO (Figure 12) to a C-terminal cysteine of ZEGFR:2377 permitted the stable ^68^Ga-labeling of this conjugate at both room temperature and 85 °C [99]. An in vivo evaluation demonstrated that [^68^Ga]Ga-DFO-ZEGFR:2377 provided a higher uptake in tumors and lower uptake in the liver compared with that using [^68^Ga]-DOTA-ZEGFR:2377 (Figure 13). This created preconditions for imaging frequently encountering hepatic metastases. The results of this and other studies [100,101] indicate that there are two molecular mechanisms of the hepatic uptake of anti-EGFR affibody molecules. One is dependent on EGFR expression by hepatocytes. Another mechanism is dependent on the distribution of charge and lipophilicity at the C-terminus of ZEGFR:2377 and its derivative ZEGFR:03115. Finding an optimal nuclide/chelator combination permits a substantial reduction in the hepatic uptake of these affibody molecules.

### 6.3. Long-Lived Positron Emitters

Clinical studies suggest that the affibody-based imaging of HER2 expression in disseminated cancer 4 h pi permits better discrimination between tumors with high and low HER2 expression than that at 2 h pi [54]. The half-life of ^68^Ga only permits this with a minimal margin. As noted above, imaging at time points beyond 3-4 h might provide a better contrast for a number of targets with expression in normal tissues. This rationalizes an exploration of long-lived positron emitters as labels for affibody molecules.

Copper-64 (T _½_ = 12.7 h, positron yield 17.8%) is an actively studied positron emitter because of its facile production using low-energy cyclotrons, commercial availability, and potential for theranostic applications [102]. One of the challenges in the application of radiocopper for imaging is its redox instability in vivo, causing the reduction of Cu(II) to Cu(I), with the subsequent release of the radionuclide from a DOTA complex and its accumulation in the liver [102,103]. In fact, all attempts using DOTA for the labeling of affibody molecules with ^64^Cu resulted in at least a two- to three-fold higher liver uptake of the conjugates in comparison with analogues labeled with ^68^Ga or ^111^In (e.g., compare data for hepatic uptake from [72] and [45], [104] and [101], [105] and [25], and [106] and [92]).

Preclinical data for short peptides suggest that the use of derivatives of triaza chelators NOTA and NODAGA provides more stable in vivo complexes with copper than DOTA, which is translated into a noticeably lower hepatic uptake [107,108]. This motivated us to evaluate NOTA and NODAGA for ^64^Cu-labeling of the synthetic ZHER2:S1 affibody molecule [109]. Surprisingly, the uptake of both variants in the liver, lungs, spleen, stomach, and bone had a clear tendency to increase with time, along with an unusually rapid decrease in the renal uptake. This phenomenon was attributed to the release into the bloodstream and redistribution of renal radiometabolites. Apparently, this effect was not noticed for other targeting peptide-based probes because of their low reabsorption in kidneys. The use of a cross-bridged CB-TE2A chelator enabled us to solve the problem of the ^64^Cu-labeling of affibody molecules [110]. It was found that the placement of a triglutamyl linker between CB-TE2A and ZHER2:S1 is essential for suppressing the initial hepatic uptake. [^64^Cu]Cu-CB-GEEE-TE2A-ZHER2:S1 provided a tumor-to-blood ratio of 185 ± 66 and a tumor-to-liver ratio of 13 ± 4 at 6 h pi.

Scandium-44 (half-life = 3.97 h, positron yield 94.3%) is a positron emitter that offers a broader imaging time window than that of ^68^Ga and can be produced using a low-energy cyclotron [111]. Although scandium and gallium are close chemical analogues, DOTA works better than NODAGA for labeling with ^44^Sc [112]. Synthetic DOTA-ZHER2:2891 was labeled with ^44^Sc at pH 4.0–4.5 in 0.5 M sodium acetate buffer for 30 min at 95 °C, with a yield of 98 ± 2% [113]. [^44^Sc]Sc-DOTA-ZHER2:2891 demonstrated a specific uptake in HER2-expressing tumors, and the tumor-to-blood ratio at 6 h pi was 51 ± 8.

Zirconium-89 (T _½_ = 3.3 days, positron yield 23.3%) is gaining increasing attention because it is essentially the only positron emitter suitable for immunoPET with full-length immunoglobulins [102]. Its use for labeling of affibody molecules might make sense when an optimal time point for imaging is the next day after injection, e.g., for EGFR-targeting affibody molecules. The use of the maleimido derivative of the DFO chelator (Figure 12) permitted nearly quantitative labeling of DFO-ZEGFR:2377 affibody molecules with ^89^Zr after one hour at room temperature [114]. A similar radiochemical yield was found for another anti-EGFR affibody clone, DFO-ZEGFR:03115 [88], and for the anti-HER3 variant DFO-ZHER3:8698 [115]. In a direct head-to-head comparison with [^89^Zr]-Zr-DFO-cetuximab, [^89^Zr]-Zr-DFO-ZEGFR:2377 demonstrated higher tumor-to-organ ratios [114]. However, the uptake in blood and bone was higher for [^89^Zr]-Zr-DFO-ZEGFR:2377 than for other EGFR-targeting affibody molecules, and this might be associated with an instability of the complex in vivo. To provide a higher label stability, another siderophore-based chelator, fusarinine C (FSC), was conjugated to ZEGFR:2377 [100]. The labeling of FSC-ZEGFR:2377 with ^89^Zr (0.5 M HEPES, pH 7, 85 °C, 30 min) resulted in a radiochemical yield of 97 ± 2%. Interestingly, the ^89^Zr-labeling of DFO-ZEGFR:2377 at 85 °C resulted in a conjugate with a lower uptake in the blood, liver, and bones than that of [^89^Zr]Zr-DFO-ZEGFR:2377 labeled at room temperature, i.e., a higher labeling temperature improved the stability of the [^89^Zr]Zr-DFO complex. The tumor-to-organ ratios for [^89^Zr]Zr-FSC-ZEGFR:2377 and [^89^Zr]Zr-DFO-ZEGFR:2377 (labeled at 85 °C) were approximately equal, but [^89^Zr]Zr-DFO-ZEGFR:2377 provided a better tumor-to-liver ratio.

The half-life of ^89^Zr might be too long for imaging the next day. Cobalt-55 (T _½_ = 17.5 h, positron yield 76%) might be a better alternative from a dosimetry point of view. This radionuclide can be produced using low-energy cyclotrons [116], and the broad availability of a long-lived surrogate ^57^Co (T _½_ = 275 d) simplifies the preclinical development of ^55^Co-labeled probes [117]. It has been demonstrated that NOTA- and DOTA-conjugated recombinant HER2-, EGFR-, and HER3-binding affibody molecules can be quantitatively labeled with ^55/57^Co by heating at 60 °C in 0.2 M ammonium acetate buffer at pH 5.5 for 30 min [51,101,118]. It was demonstrated that the tumor-to-blood and tumor-to-lung ratios for [^57^Co]Co-NOTA-HEHEHE-ZHER3:06898 are 1.5-2-fold higher at 24 h than at 3 h, and this could improve the sensitivity of imaging [51]. [^57^Co]-Co-DOTA-ZEGFR:2377 provided the best tumor-to-liver ratio among all tested affibody-based EGFR imaging probes [101] (Figure 13).

## 7. Conclusions

We believe that the following considerations should be taken into account for the preclinical and clinical development of affibody-based imaging probes:

- Affinity should be in the low nanomolar range for targets with a high expression, and a subnanomolar affinity is necessary in the case of low expression;
- A desirable affinity should be achieved by affinity maturation, not by dimerization;
- Meticulous dose-finding studies should be performed in the case of the physiological expression of a target in normal tissues. Conventional microdosing studies might be misleading due to a much higher normal tissue uptake when compared to imaging using optimal doses;
- The molecular design of affibody-based tracers should aim to minimize off-target interactions. Labeling approaches resulting in an increased lipophilicity are undesirable, but their negative effect might be compensated for by adding a negatively charged linker (e.g., a triglutamyl linker).
- The combination of a radiometal and chelator has an appreciable influence on off-target interactions and thus on the imaging contrast. Several alternative approaches should be tested to select the best one.
